# Th17 cytokine deficiency in patients with *Aspergillus* skull base osteomyelitis

**DOI:** 10.1186/s12879-015-0891-2

**Published:** 2015-03-21

**Authors:** Corine E Delsing, Katharina L Becker, Anna Simon, Bart Jan Kullberg, Chantal P Bleeker-Rovers, Frank L van de Veerdonk, Mihai G Netea

**Affiliations:** Department of Internal Medicine and Radboudumc Center for Infectious Diseases, Radboud University Medical Center, Geert Grooteplein Zuid 8, 6525 GA Nijmegen, The Netherlands

**Keywords:** *Aspergillus*, Skull base osteomyelitis, Interleukin-17, Interleukin-22, Th17 response, Antifungal host defense

## Abstract

**Background:**

Fungal skull base osteomyelitis (SBO) is a severe complication of otitis externa or sinonasal infection, and is mainly caused by *Aspergillus* species. Here we investigate innate and adaptive immune responses in patients with *Aspergillus* SBO to identify defects in the immune response that could explain the susceptibility to this devastating disease.

**Methods:**

Peripheral blood mononuclear cells isolated from six patients with *Aspergillus* SBO and healthy volunteers were stimulated with various microbial stimuli, among which also the fungal pathogens *Candida albicans* and *Aspergillus fumigatus.* The proinflammatory cytokines IL-6, TNFα and IL-1β, and the T-helper cell-derived cytokines IFNγ, IL-17 and IL-22 were measured in cell culture supernatants by ELISA.

**Results:**

Proinflammatory cytokine responses did not differ between SBO patients and healthy volunteers. The *Candida*- and *Aspergillus*-specific Th17 response (production of IL-17 and IL-22) was significantly decreased in the SBO patients compared to healthy individuals, while Th1 cytokine response (IFNγ production) did not differ between the two groups.

**Conclusions:**

We show that patients with *Aspergillus* skull base osteomyelitis infection have specific defects in Th17 responses. Since IL-17 and IL-22 are important for stimulating antifungal host defense, we hypothesize that strategies that have the ability to improve IL-17 and IL-22 production may be useful as adjuvant immunotherapy in patients with *Aspergillus* SBO.

## Background

Skull base osteomyelitis (SBO) is a rare but life-threatening infection, which originates either from the external ear canal or the paranasal sinus. Infiltrative growth from the external acoustic duct into the temporal bone is also termed malignant or invasive otitis externa [1]. This aggressive infection is usually caused by *Pseudomonas aeruginosa*, sometimes by fungal pathogens (mainly *Aspergillus* spp.)*,* and in a minority of cases by other bacteria (such as S*taphylococci*, *Proteus* and *Klebsiella*) [2-4]. Besides an otologic origin of infection, sinusitis is the second major cause of skull base osteomyelitis. Although this is a rare complication, infections of the frontal, ethmoid, sphenoid and maxillary sinus can spread to the orbital and frontal bone, clivus and petrous apices. A distinct form of sinusitis is fungal rhinosinusitis. This has a broad clinical spectrum ranging from chronic forms with gradually progressing osteomyelitis to necrotizing angioinvasive disease. *Aspergillus, Rhizopus* and *Fusarium* are the most commonly identified fungi. Fungal skull base osteomyelitis due to *Aspergillus* is an infection with considerable morbidity and mortality rates up to 50%. In addition to aggressive surgical debridement and systemic antifungal therapy, the mainstay of therapy includes, whenever possible, correction of the underlying immunologic defect.

Spores of *Aspergillus* are continuously inhaled and therefore fungal colonization of the upper airways is common. Despite this continuous exposure, invasive disease caused by *Aspergillus* in an immunocompetent host is very rare. Although some degree of immunosuppression may be present in patients with fungal SBO, often the only apparent risk factor identified is a chronic external otitis or an anatomical obstruction of the sinuses (e.g. nasal polyps or chronic inflammation of the mucosa). The extent of tissue invasion in these patients may vary depending on the underlying immune status of the host. In the present study we present six cases of skull base osteomyelitis due to *Aspergillus fumigatus* and *Aspergillus flavus*, in whom we investigated whether the innate and adaptive immune responses known to be important for antifungal host defense are defective.

## Methods

### Volunteers and patients

We describe six patients who were admitted to the Radboud University Medical Center with a culture-proven invasive *Aspergillus* osteomyelitis of the skull. All patients were diagnosed between September 2007 and July 2010. Charts were reviewed for data on demographics, risk factors, presenting symptoms, treatment, side effects, microbiology results, and clinical outcome. Response was defined according to the revised MSG/EORTC consensus group definition [5]. Patients and healthy volunteers, who served as healthy controls in the immunological experiments, were asked for blood donations. The blood samples were collected from patients and healthy volunteers after informed consent was obtained in accordance to Good Clinical practice, the Declaration of Helsinki, and the approval of the Arnhem-Nijmegen Ethics Committee (nr.2010/104).

### Stimuli

*E.coli* lipopolysaccharide (LPS) (10 ng/ml) (TLR4 ligand, *E. coli* serotype O55:B5, Sigma-Aldrich St. Louis, MO USA); heat-killed *Staphylococcus aureus* clinical isolate (*S. aureus*) (1×10^7^/ml); heat-killed *Candida albicans* yeast ATCC MYA-3573 (UC820) (*C. albicans*) (1×10^5^/ml); heat-killed *Aspergillus fumigatus* clinical isolate V05-27 (*A. fumigatus*) conidia (1×10^7^/ml) were cultured and isolated as described previously [6].

### Peripheral blood mononuclear cells (PBMCs) isolation

Fresh venous blood was drawn in 10 ml EDTA tubes from patients and controls and processed in parallel continuously from PBMC isolation to cytokine measurement. Every patient gave blood only once. The blood was diluted 1:1 with Phosphate Buffered Saline (PBS). Subsequently PBMCs were isolated using Ficoll-paque (GE Healthcare, Zeist, The Netherlands) density gradient centrifugation. The PBMCs layer was collected and washed twice in cold PBS. Cells were reconstituted in RPMI-1640 culture medium (Dutch modification, Gibco, Invitrogen, Breda, The Netherlands) supplemented with 10 μg/ml gentamicin, 10 mM L-glutamine and 10 mM pyruvate (Gibco). The cells were counted with a particle counter (Beckmann Coulter, Woerden, The Netherlands) and the concentration was adjusted to 5×10^6^ cells/ml.

### PBMCs stimulation

PBMCs were plated in a 96-well plate (Corning, NY, USA) at a final concentration of 2.5×10^6^/ml in an end volume of 200 μl per well. Either medium or stimuli were added. Cells were incubated at 37°C with 5% CO_2_, after 24 or 48 hours or 7 days supernatants were collected and stored at −20°C. Seven-day stimulations were performed in the presence of 10% pooled human serum. All stimulations assays were performed in duplicates.

### Cytokines measurements

Cytokines were measured in the cell culture supernatants using a commercial ELISA kit (IL-1β, TNFα, IL-17 and IL-22: R&D Systems; IL-6 and IFNγ: Sanquin) according to the instructions supplied by the manufacturer. Proinflammatory cytokines production was measured after 24 hours, IFNγ after 48 hours and the T helper cytokines IL-22 and IL-17 after 7 days of incubation.

### Statistical analysis

The Mann–Whitney-*U* test was used to detect differences between healthy controls and patients. A *p*-value of < 0.05 was considered statistically significant (* = *p* < 0.05, ** = *p* < 0.01 and *** = *p* < 0.001). Graphs represent cumulative results of all performed experiments and are presented as mean ± standard error of the mean. Data were analyzed with GraphPad Prism v 5.0.

## Results

### Demographic characteristics of patients and controls

#### Patients

Three male and three female patients were included in the study with age ranging from 37 to 87 years (mean: 59.5 years). Infections were located in the sphenoid, mastoid or ethmoid bones with partial affection of sinus cavernosus, frontal or temporal lobe or orbita. All patients presented with cranial nerve palsy. In four cases the diagnosis was additionally confirmed by a positive histology. One patient developed *Aspergillus* osteomyelitis following trans-sphenoidal surgery for pituitary adenoma with a chronic myeloid leukemia (CML) in the past; two had a history of diabetes; all other patients had primary fungal infection of sinus or mastoid.

#### Controls

Three male and three female healthy volunteers were included in the control group; the age ranged from 24 to 60 years (mean: 42.7 years). Three of the patients were recruited from the blood bank via the blood donor service (Sanquin, Nijmegen, The Netherlands) and three were recruited directly to our department for blood donation. All volunteers were healthy and did not have an immunologically relevant medical history.

### Microbiology

Culture and molecular identification confirmed the fungal infection and susceptibility to antifungal drugs was tested (Table 1). Galactomannan assay was performed in all patients on serum and in 4 patients on cerebrospinal fluid. All results were negative (index < 0.5).

**Table 1:** **Anti-fungal susceptibility of microbiological isolates**

**Case**	**Isolate**	**AMT (mg/L)**	**ITC (mg/L)**	**VOR (mg/L)**	**Anidula (mg/L)**	**POSA (mg/L)**	**CASPO (mg/L)**
**1**	*A. fumigatus*	*					
**2**	*A. fumigatus*	1	1	2	0.063	0.25	
**3**	*A. fumigatus*	0.5	0.063	0.125		< 0.016	0.5
**4**	*A. fumigatus*	1	0.25	1	0.031	0.063	
**5**	*A. fumigatus*	**					
**6**	*A. flavus*	1	0.063	1		0.031	0.5

*MIC impossible because of poor sporulation. Analysis of Cyp51A-gene: no TR/L98H.

**No isolate available for susceptibility testing.

MIC values of fungal isolates tested for Amphotericin B (AMT), Itraconazole (ITC), Voriconazole (VOR), Anidulafungin (Anidula), Posconazole (POSA) and Caspofungin (CASPO) are listed.

### Treatment, clinical outcome and blood sampling

Surgical debulking was performed in all patients. All patients were initially treated with systemic antifungal drug therapy. Voriconazole was first line treatment in all patients. Four patients were treated with voriconazole monotherapy. One patient was concomitantly treated with liposomal amphotericine B during the first months of treatment. Posaconazole was used after induction treatment in two patients. Duration of therapy (from 4.5 to 35 months) was guided by clinical response and imaging, with follow-up from 8 to 38 months. Four patients had a complete response, one had a relapse, and one died due to respiratory failure. During infection the leukocytes count was normal in five of six patients, ranging from 6.6 × 10^9^/l to 9.1 × 10^9^/l. One patient had a decreased leukocyte count of 3.1 × 10^9^/l and slightly decreased numbers in the differential blood count. Three patients had normal and three patients had increased CRP values (11 mg/l, 14 mg/l and 32 mg/l). Blood samples for immunological assays were taken after clinical improvement and response to the therapy: in three patients during the first month after onset of the antifungal therapy, while in three patients blood was collected after the pharmacological therapy had been finished and the infection did not recur.

### *Patients with* Aspergillus *SBO do not differ from healthy controls in their production of proinflammatory cytokines*

**Figure 1:**
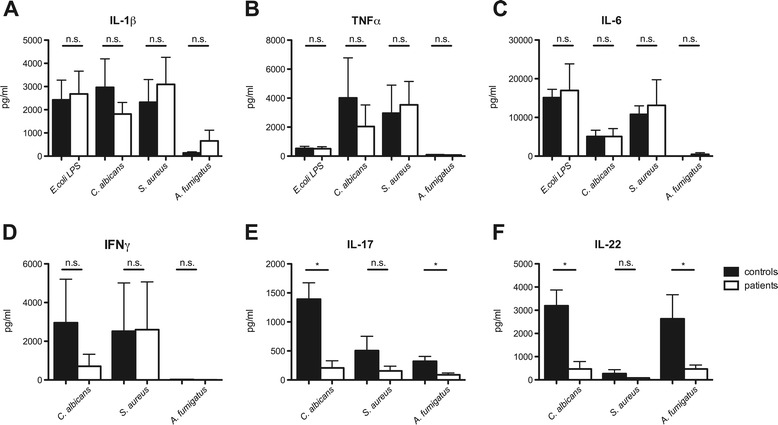
**SBO patients have an intact innate immune response, but are defective in IL-17 and IL-22 production.** PBMCs stimulated with *E. coli* LPS, heat killed *C. albicans* yeast, *S. aureus* and *A. fumigatus* were cultured for 24 hours 48 hours or 7 days respectively. The innate cytokines IL-1β **(A)**, TNFα **(B)**, IL-6 **(C)** (after 24 hours) and adaptive cytokines IFNγ **(D)** (after 48 hours) and IL-17 **(E)** /IL-22 **(F)** (after 7 days) were measured in the cell culture supernatant by ELISA. Controls (black bars, n = 6) compared with SBO patients (white bars, n = 6).

To investigate the innate immune response, PBMCs isolated from six patients with *Aspergillus* SBO were stimulated with *Aspergillus fumigatus*, *E. coli* LPS and different pathogens and compared with healthy controls (Figure 1 A-C). Neither the unspecific LPS-stimulation, nor the pathogen-specific *C. albicans, S. aureus,* and disease-specific *A. fumigatus* stimulations showed differences in the production of the inflammatory cytokines IL-1β, TNFα and IL-6.

### *Patients with* Aspergillus *SBO are deficient in* Aspergillus*-induced IL-17 and IL-22 but not in IFNγ production*

While the recognition and initiation of inflammatory cytokine responses revealed to be intact, we wanted to address the question whether defects in the acquired immune response might explain the high susceptibility to the fungal infection of the six fungal SBO patients included in this study. PBMCs were stimulated with *C. albicans* and *S. aureus*, serving as positive controls for the induction of IFNγ, IL-17 and IL-22, and *A. fumigatus*, to investigate pathogen-specific deficiencies. The Th1 response, shown by IFNγ production, did not differ between patients with *Aspergillus* SBO and healthy controls (Figure 1D). In contrast, IL-22 and IL-17 production was significantly reduced after stimulation with both fungal pathogens *C. albicans* and *A. fumigatus* (Figure 1E-F).

## Discussion

In this study, we describe the clinical presentation and immunological features of six patients with *Aspergillus* SBO. None of the patients were neutropenic at the time of the infection or had a known primary immunodeficiency. We hypothesized that specific defects in *Aspergillus*-specific innate and/or adaptive immune response would contribute to the unsuccessful fungal clearance and extent of the *Aspergillus* infection in our patients. Therefore, we investigated host responses in six patients with *Aspergillus*-SBO. While the innate responses were not different from a healthy control group, Th17 cytokines induced by fungal pathogens such as *C. albicans* and *A. fumigatus* were shown to be defective in the patients with *Aspergillus* SBO.

*Aspergillus* spp. can cause several forms of diseases dependent on the site of infection and immune status of the host. *Aspergillus* osteomyelitis is increasingly being reported [7] with approximately 15% of the cases affecting the skull base [7,8]. *Aspergillus* SBO is a severe complication of otitis externa or invasive sinonasal aspergillosis, in which most patients become infected via the tympanic cavity or the sinus [2].

The main risk factors described for invasive *Aspergillus*-SBO are systemic immunosuppression [9] and hematologic malignancies (12%) [7,10]. One patient included in this study suffered from chronic myeloid leukemia, and he was treated with the tyrosine kinase inhibitor imatinib at the time of the infection. Two patients had diabetes mellitus and three suffered from sinusitis, which have been reported in previous studies as risk factors [7,10]. Although almost all patients had normal leukocyte counts (one was slightly decreased), additional factors influencing the immune response such as the imatinib treatment, diabetes or the high age of one patient might affect the IL-17 response as well. Normal monocyte-derived cytokine levels point to a specific T-cell defect.

Early recognition and therapeutic intervention in invasive sinonasal aspergillosis with systemic antifungal therapy and surgical resection and/or debridement is important. In accordance with the current guidelines, all patients included in the present study were treated with surgical debridement of the infected bone and systemic antifungal drug therapy, of which voriconazole was the drug of first choice. We observed a mortality of 1 out of 6 in the patients included in the present study, which is in line with the reported poor clinical outcome of *Aspergillus* osteomyelitis, which has a 25% 12-weeks mortality [11].

Why did our patients without an apparent severe immunodeficiency get invasive aspergillosis? *Aspergillus* spp*.* are an occasional commensal of the external ear and paranasal sinuses [12], but invasive disease is very rare. Chronic infection leading to damage of the epithelial barrier is an important entry for the fungus to infect the host. Recognition of *Aspergillus* will result in the production of proinflammatory cytokines that will recruit immune cells to clear the infection [13]. In the present study, patients with *Aspergillus* SBO showed normal production of the cytokines TNFα, IL-1β and IL-6 after stimulation with *A. fumigatus*, *C. albicans*, the Gram-positive bacterium *S. aureus*, and the Gram-negative cell wall component *E. coli* LPS*.* Therefore, a defect in the production of monocyte-derived proinflammatory cytokines is unlikely to be the cause of the susceptibility of fungal SBO in our patients. Acquired adaptive T-helper responses also play an important role in anti-*Aspergillu*s host defense. The protective role of IFNγ in the *Aspergillus*-specific host response has been reported previously [14]. We did not observe a difference in *Aspergillus*-specific IFNγ production; *Candida*-specific IFNγ production showed a trend towards a lower IFNγ production, but this was not significant. However, the *Aspergillus*-specific Th17 response was significantly lower in SBO patients compared to the healthy control group. In addition, this was also observed, when the cells were stimulated with *Candida.* IL-17 is a characteristic cytokine produced by Th17 cells. Th17 cells are crucial for neutrophil recruitment and controlling fungal invasion at the level of mucosae and skin [15]. Similar to IL-17, the IL-22 production was significantly decreased in SBO patients compared to healthy controls in the present study. IL-22 is also produced by Th17 cells [16] and shares many effector functions with IL-17 [17]. IL-22 plays a predominant role in mucosal host defense [18] by inducing anti-microbial peptides produced by epithelial cells, which can kill microorganisms directly [19].

In a previous study we have identified that IFNγ treatment had beneficial effects on the immune status including IL-17 and IL-22 responses in a case series of patients with invasive fungal infections [20]. This suggests that adjuvant therapy with recombinant IFNγ may improve the outcome of patients with a severe fungal SBO. Another treatment option would be to increase the differentiation into Th1 and Th17 cells by GM-CSF [21]. GM-CSF was shown to enhance the secretion of inflammatory cytokines [22] and antigen-presentation [23] under inflammatory conditions. It also induces the differentiation of progenitors cells into monocytes and granulocytes [24]. Therefore, GM-CSF might have beneficial effects on immune cells in the skin even in the setting of an IL-17/IL-22 deficiency [25]. One might speculate about supplementary therapy with recombinant IL-22, since IL-22 was shown to have beneficial effect in wound healing processes and pre-clinical studies have shown good toleration of administration of the drug [26].

We are aware of the fact that the sample size of six patients is inevitably small. A type I error of 5%, meaning the likelihood to accept the hypothesis that *Aspergillus* SBO patients and control do not differ in their *Aspergillus*-specific IL-17 response was assumed. Using the calculated means, standard deviations of the experimental measurements and sample size of both groups we calculated a statistical power of 81%. Thus, the Type II error, meaning the hypothesis that patients and controls differ in their IL-17 response is neglected, lay with 19% in an acceptable range for medical tests. Retrospectively, due to the differences observed, the sample size of 6 donors was the minimal size needed to detect statistically relevant differences. Further sample size independent calculation revealed an effect size of 1.5, which indicates that *Aspergillus* SBO has a statistically strong effect on the *Aspergillus*-specific IL-17 production.

Nevertheless, there are also some limitations of the study. One note of caution is that while patients and controls matched regarding their gender, the patients were in average older, although the 15 years difference is unlikely to explain the significant differences observed. Further, it remains unknown whether the defective IL-17 and IL-22 production was the consequence of a primary defect (e.g. genetic) or was secondary to a predisposing factor of the *Aspergillus* SBO patients (e.g. the antifungal treatment). Nevertheless, the present study contributes to understanding the specific defective host defense mechanisms underlying SBO due to *Aspergillus*.

## Conclusions

This is the first study describing a deficiency in fungal-induced Th17 responses in patients with *Aspergillus* skull base osteomyelitis. Future studies are needed to validate this observation and its clinical implication, especially the potential beneficial effects of immunotherapy aimed to boost Th17 responses.
